# A global review of mistletoe frugivory and seed dispersal: The plant perspective

**DOI:** 10.1111/plb.70252

**Published:** 2026-06-29

**Authors:** R. F. Fadini, M. A. Pizo, F. E. Fontúrbel, E. Cazetta

**Affiliations:** ^1^ Laboratório de Ecologia e Conservação (LabECon) Instituto de Biodiversidade e Florestas, Universidade Federal do Oeste do Pará Santarém Brazil; ^2^ Laboratório de Ecologia Aplicada à Conservação (LEAC) Universidade Estadual de Santa Cruz Ilhéus Brazil; ^3^ Grupo de Estudos em Plantas Parasitas Universidade Estadual do Sudoeste da Bahia Vitória da Conquista Brazil; ^4^ DISPERSE: Rede de Pesquisa sobre Frugivoria e Dispersão de Sementes Universidade Estadual de Santa Cruz Ilhéus Brazil; ^5^ Laboratório de Ecologia de Aves Universidade Estadual Paulista Júlio de Mesquista Filho Rio Claro Brazil; ^6^ Instituto de Biología, Pontificia Universidad Católica de Valparaíso Valparaíso Chile; ^7^ Millennium Nucleus of Patagonian Limit of Life (LiLi) Valdivia Chile; ^8^ Laboratório de Ecologia de Interações (LABINT) Universidade Estadual de Santa Cruz Ilhéus Brazil

**Keywords:** Birds, endozoochory, Loranthaceae, parasitic plants, Santalaceae, study gaps

## Abstract

Mistletoe–frugivore interactions stand out as one of the classic examples of specialized and reciprocal relationships in ecology and evolutionary biology. Additionally, many theoretical advances have been made by using the interactions between mistletoes and their seed dispersers as study models. Although generally well‐studied, there is no single global review on mistletoe frugivory and seed dispersal, creating an opportunity to synthesize existing knowledge, identify major gaps, and advance unresolved and new research questions. We conducted a systematic review of studies on mistletoe frugivory and seed dispersal, focusing on taxonomic, geographic, and methodological patterns, showcasing the plant perspective, which has been comparatively underappreciated relative to seed dispersers' perspective. The number of studies (n = 58) and species studied (n = 36) was proportional to the number of species per genus, although several diverse genera remain understudied. We found very few studies in the tropical rainforests and in the north temperate forests. No endangered mistletoe species (n = 58) have been studied, and only one species has been studied in urban areas. Methodologically, searching for seeds or seedlings in potential recruitment sites predominated, while tracking movements of seed dispersers with radio or GPS devices remains poorly used. Mistletoe–frugivore interactions offer excellent study models for exploring a different suite of ecological questions, yet substantial gaps persist across taxa, regions, and biomes. Future studies should prioritize rarer species, especially those threatened and endemic. Studies should also prioritize understudied biomes, such as the tropical rainforests and north temperate forests. We further believe that many unanswered research questions could be clarified using appropriate and modern methods to assess and record interactions between mistletoes and frugivores, thereby advancing the use of these systems as model frameworks for studying the ecological and evolutionary consequences of seed dispersal.

## Graphical abstract

We reviewed studies on mistletoe frugivory and seed dispersal worldwide. Although mistletoes are relatively well studied, major taxonomic, geographic, and methodological gaps remain. We conclude that modern technologies offer new opportunities to address long‐standing but still poorly explored questions about these interactions.

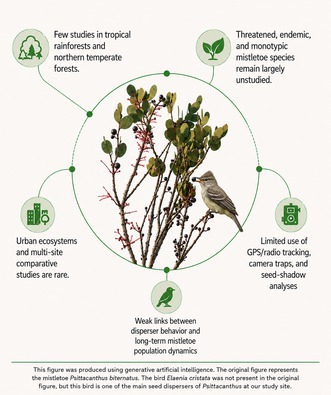

## INTRODUCTION

Mistletoes are a diverse group of aerial hemiparasitic plants that comprise ca. 1,600 species of worldwide distribution (except in the poles) (Nickrent 2011). Growing on the top of tree canopies because of their light demands, mistletoes absorb water and nutrients from their hosts and reproduce profusely. As a group, mistletoes are considered pests of orchards, forest plantations, and cities, but a few species are threatened to some degree (Fadini *et al*. 2024), and several others provide food (fruits, flowers, and leaves), nesting support, and shelter for a diverse assemblage of animals year‐round (Watson 2001). Additionally, mistletoe plants produce a nutrient‐rich litter, which is readily absorbed by non‐host plants. Therefore, mistletoes are considered keystone species, whose effects permeate scales and levels of organization, influencing the fitness of individuals, the persistence of populations, and the structure of communities (Griebel *et al*. 2017; Watson *et al*. 2022).

Among the many ecological interactions involving mistletoes, those established with frugivorous birds stand out as one of the classic examples of specialized and reciprocal relationships in ecology and evolutionary biology, dating back to Darwin's On the Origin of Species. Additionally, many advances in ecological and evolutionary theories have been made by using these interactions as study models. In particular, studies on mistletoes and their seed dispersers have been central to evolutionary explanations for seed dispersal, including the formulation of the directed dispersal hypothesis (Green *et al*. 2009). Mistletoe–frugivore interactions have also a central role in the development of ecological approaches, such as the Seed Dispersal Effectiveness Framework (SDE) (Montaño‐Centellas 2013), in theories addressing the persistence of species across space, such as the metapopulation theory (Overton 1994), and in shaping our understanding of parasite transmission dynamics (Martínez del Rio *et al*. 1996).

Early work on mistletoe–frugivore interactions focused largely on the morphophysiological adaptations of birds for consuming mistletoe fruits, with classic studies documenting species groups such as euphonias feeding on *Phoradendron* Nutt. and *Dendrophthora* Eich. (Wetmore 1914), *Euphonia elegantissima* and *Bombycilla cedrorum* feeding on *Phoradendron* (Sutton 1951), and *Phainopepla nitens* consuming *Phoradendron californicum* Nutt. (Walsberg 1975). These foundational studies were followed by detailed natural history observations and more naturalistic syntheses, particularly in the tropics (Snow 1981) and Europe (Snow & Snow 1984). Notably, information on mistletoe–frugivore interactions in tropical regions, including tropical Asia (*e.g., Dicaeum* and Loranthaceae), dates back even earlier (citations in van Docters 1954). In the Neotropics, more quantitative information emerged later, with pioneering studies by Carla Restrepo (Restrepo 1987; Restrepo *et al*. 2002), and Sarah Sargent (Sargent 1994), documenting in detail the frugivory and seed dispersal of diverse mistletoe species and genera (*Phoradendron* and *Andidaphne* Poepp. & Endl., both Santalaceae, and *Cladocolea* Tiegh., Loranthaceae).

While global reviews on mistletoe ecology have been published over the past decades (Watson 2001; Watson *et al*. 2022 – exploring, for example, the keystone role of mistletoes as resources; Mathiasen *et al*. 2008 – basic ecological and evolutionary history of mistletoes; and Griebel *et al*. 2017 – positive and negative impacts of mistletoes on ecosystem properties), to the best of our knowledge, no global synthesis has focused specifically on mistletoe frugivory and seed dispersal. Here, we fill this gap by providing a systematic review centred on the plant's perspective, which has been comparatively underexplored relative to the seed disperser's perspective (Restrepo *et al*. 2002; Watson 2025).

We explored three facets of mistletoe frugivory and seed dispersal: taxonomic, geographic, and methodological. From a taxonomic perspective, we synthesize current knowledge on interactions between frugivores and mistletoes and assess whether research effort and species coverage track genus‐level mistletoe diversity. Geographically, we evaluated the distribution of studies across regions, vegetation types, and conservation status of the studied species, with particular attention to persistent biases against tropical forests in studies of mistletoes and their frugivory and seed dispersal (Watson 2004). Methodologically, we review the methods and response variables most commonly used to quantify the consequences of seed dispersal for mistletoe performance and identify emerging strategies and new questions that need to be addressed. Beyond synthesizing existing knowledge, our aim is to provide new perspectives and ideas for the future generation of ecologists who study mistletoe and their interactions with frugivores and seed dispersers, as well as making all the information obtained available for researchers.

## METHODS

We conducted a systematic review on 24 June 2025, without time restrictions, using the Web of Science and Scopus databases. Searches were performed using a combination of the following terms: Frugivor* AND mistletoe, ‘Seed dispers*’ AND mistletoe, Dispers* AND mistletoe, Frugivor* AND Loranthaceae, Frugivor* AND Santalaceae (Loranthaceae and Santalaceae are the most diverse mistletoe families). We also searched for outdated mistletoe family names (Amphorogynaceae, Eremolepidaceae, and Viscaceae), but excluded Misodendraceae because this family lacks species with fleshy fruits. All searches were conducted in English. We also performed searches in Portuguese and Spanish using the Google Scholar platform, but these yielded no additional records and were therefore excluded from the review.

Our main interest was to include studies with a clear focus on mistletoe frugivory and seed dispersal that presented at least some quantitative information, mainly focused on both the quantity (number of visits and number of seeds dispersed per visit) and quality (quality of seed treatment and quality of seed deposition) components of seed dispersal (*sensu* Schupp 1993; Schupp *et al*. 2010). Studies with focus on animals and mentioning mistletoes only tangentially as part of a broader diet were not considered (*e.g*., Carlo *et al*. 2004). A complete list of the inclusion/exclusion criteria is provided in the Table S1. Data were collected following the PRISMA protocol (PRISMA‐EcoEvo 1.0; O'Dea *et al*. 2021). For each selected article, we recorded basic information (*e.g*., article ID, publication year, authors, title, study site geographical coordinates, country, mistletoe and frugivore genus and species studied, etc.). Then, we categorized the vegetation type of each study site in Forest, Savanna, Desert or Semi‐desert, and Shrubland, based on the information available in the Methods session of each article. Finally, we collected detailed information on the methods and response variables used to record mistletoe–frugivore interactions and their immediate consequences for plants (*i.e*., seed rain or seedling establishment).

## RESULTS

**Fig. 1:**
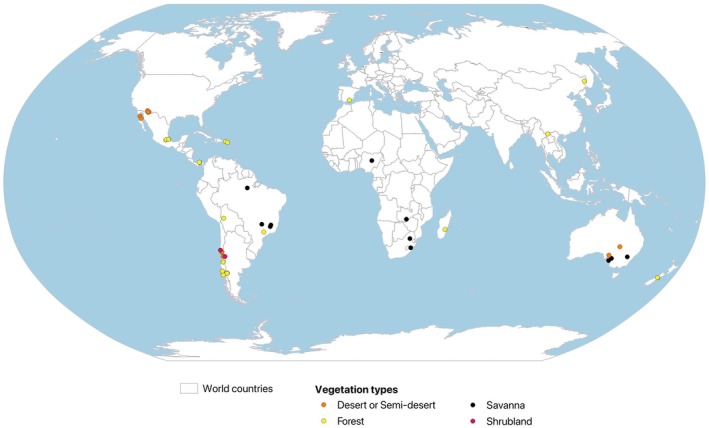
Location of the 58 studies used in this review. The colour indicates the vegetation type where the study was conducted with information from the methods section.

**Fig. 2:**
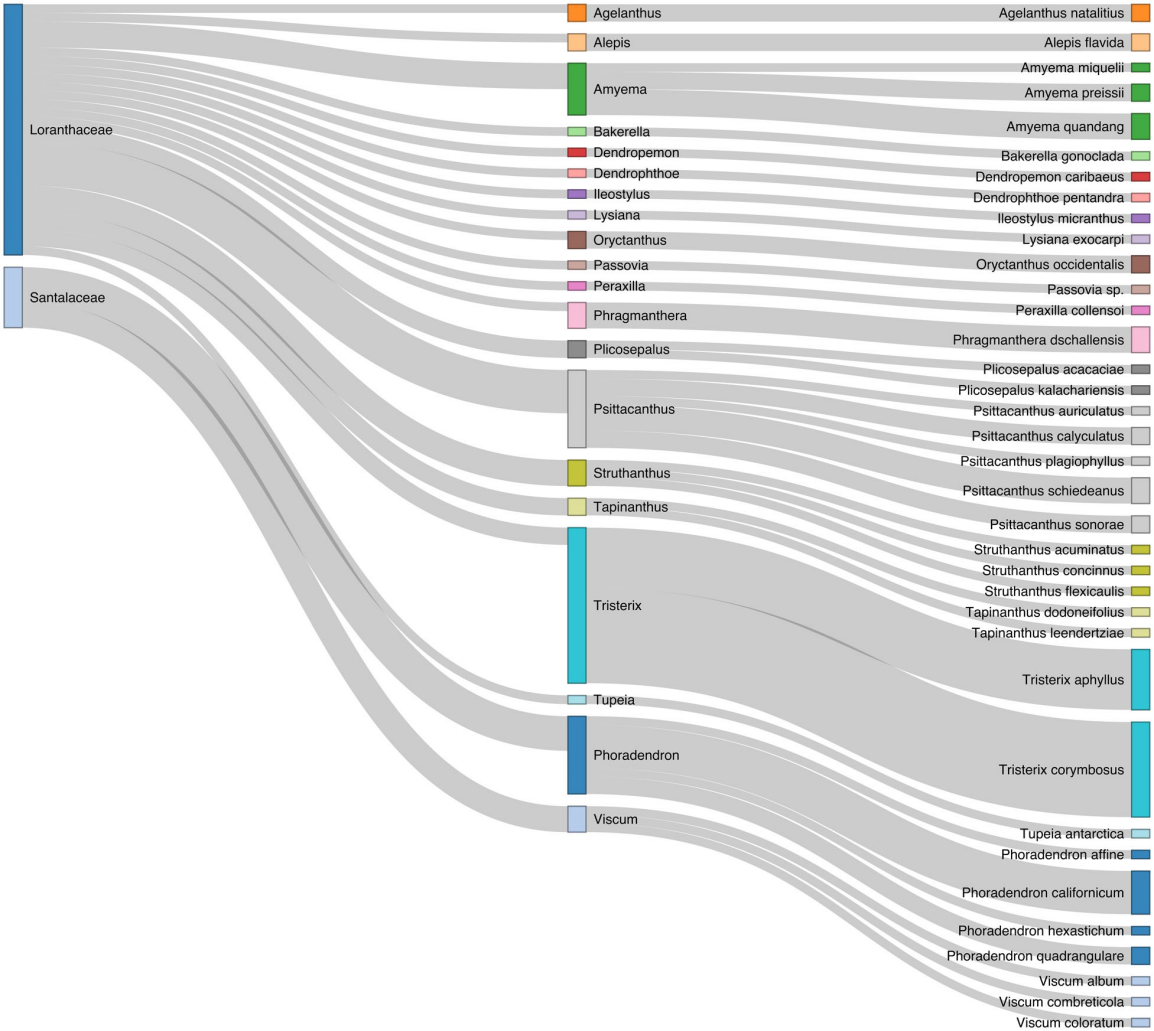
Sankey diagram showing the number of studies conducted for each mistletoe family, genus, and species. The height of the column represents the number of studies. See the scale at the bottom of the figure. N = 58 studies.

Our review resulted in 58 articles (Fig. S1) that provided 73 case studies. The number of case studies were higher than the number of articles because some studies investigated more than one mistletoe species or biome (Data S1). Studies accumulate through time from 1983 to 2025, but deaccelerate in the last decade (Fig. S2). Studies covered 36 mistletoe species and 20 genera spread through 17 countries (Figs 1 and 2). Chile was the country with the majority of studies (11, ~20% of all studies). Within Loranthaceae, the reviewed studies covered approximately 25% of the genera and about 3% of the species assessed. Within Santalaceae, they encompassed about 25% of the genera and about 1.3% of the species bearing fleshy fruits (*i.e*., excluding *Arceuthobium* M.Bieb. and *Korthalsella* Tiegh.). The number of studies and the number of species studied per genus were positively correlated with the number of species per genus (Pearson's correlation R = 0.50 and 0.72, respectively, *P* < 0.05 in both cases).

About 45% of the studies were conducted exclusively in forests, 28% in deserts or semi‐deserts, 22% in savannas, and 2% in shrublands. A smaller proportion of studies spanned more than one vegetation type, with 6% in savannas and deserts/semi‐deserts, and 3% in forests and shrublands. The genus *Tristerix* Mart., in temperate South America, was the most studied and *T. corymbosus* L. Kuijt, occurring both in the Chilean matorral (Shrubland) and in the Temperate Forests of South America, was the most studied species (n = 11 studies) (Fig. 2). The most diverse genera within the Loranthaceae and Santalaceae, *Psittacanthus* Mart. and *Phoradendron*, respectively, had nine studies each. We found very few studies conducted in temperate forests of the north and, surprisingly, only one study of the European mistletoe *Viscum album* L. No studies were found for *Loranthus europaeus* Jacq. either, despite the fact that both species are common in Europe.

Most studies (86%) were conducted on a single mistletoe species (range: 1–4) and study site (~85%, range: 1–22). Average number of frugivores interacting with mistletoe species was 2.7 ± 2.1 per study (range: 1–11). Twenty‐eight animal families (26 of birds and 2 of mammals), of 49 genera, and 65 species were recorded eating fruits of Loranthaceae, while 16 bird families, of 24 genera, and 32 species consumed fruits of Santalaceae (Data S1). No mistletoe species listed in the IUCN redlist have been studied, nor have any species been studied in mature tropical rainforests, the most diverse mistletoe biome. Only one study was conducted in an urban area (*Phoradendron affine* (Pohl ex DC.) Engl. & Krause, Santalaceae).

**Fig. 3:**
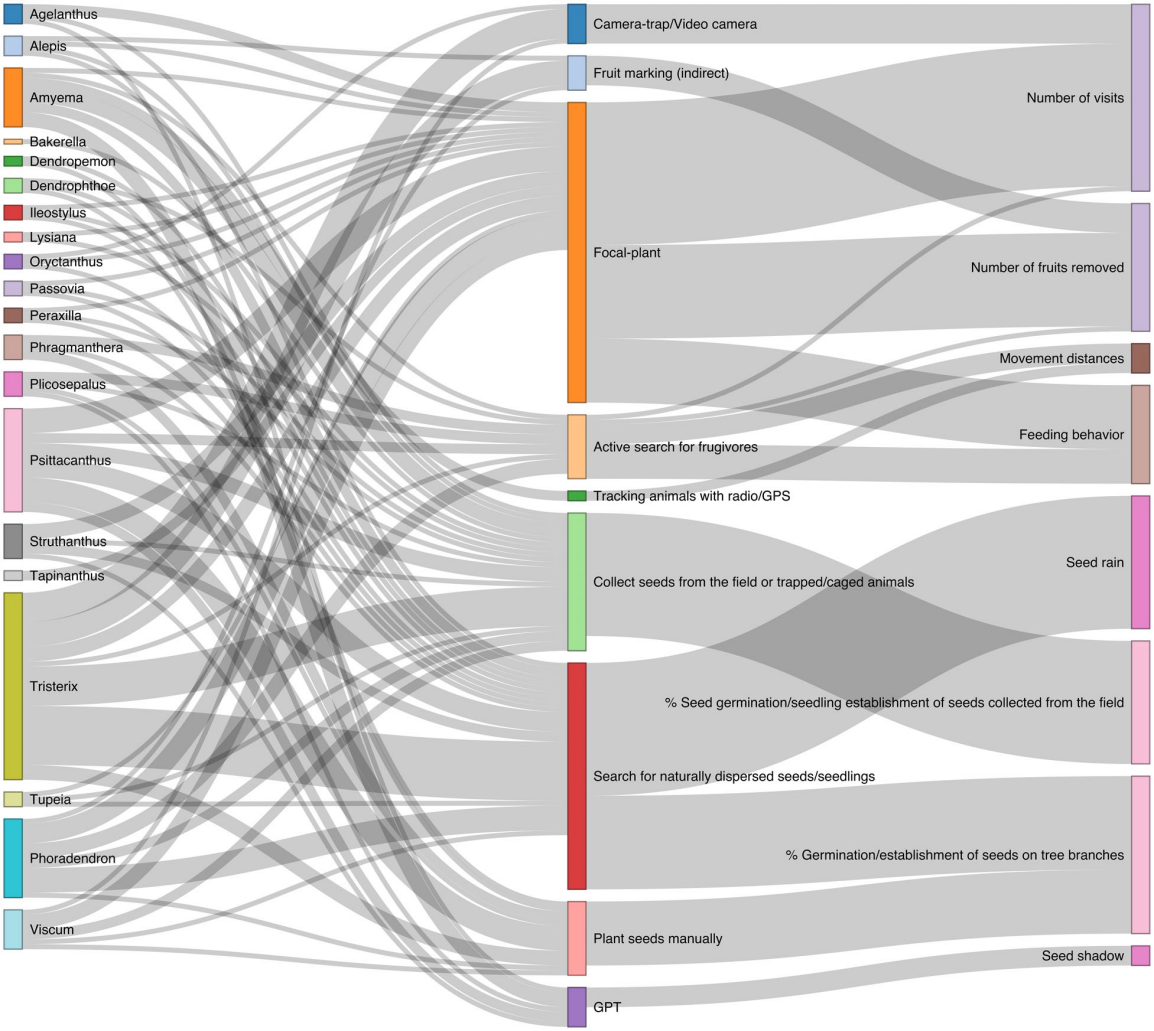
Sankey diagram showing the number of cases using each method and response variable to study mistletoe‐frugivore interactions. N = 58 studies and 73 cases.

Regarding methodological approaches, searching for seeds or seedlings in potential recruitment sites (*e.g*., tree branches) was the most used method (n = 35 case studies), followed by the classical focal‐plant method to record mistletoe–frugivore interactions (n = 30) (Table 1 and Fig. 3). Studies frequently used on average two methods (range: 1–5). Tracking movements of seed dispersers with radio or GPS devices, recording fruit removal indirectly, and using camera traps or video cameras to record mistletoe–animal interactions were rarely used in comparison to other methods (n = 2, 7, and 8 case studies, respectively). The number of visits and the percentage of germination or establishment of seeds on tree branches were the most frequently used response variables (n = 34 and 29 case studies, respectively), while ‘seed shadow’ was underrepresented (n = 4). Fifteen percent of cases used response variables to quantify only the quantity component of the seed dispersal, 45% only the quality component, and 40% of the cases used at least one response variable to characterize the quantity and the quality components simultaneously (*sensu* Schupp 1993; Schupp *et al*. 2010).

**Table 1:** Methods and response variables used to study mistletoe–frugivore interactions obtained from the 58 studies consulted for this review.

type/short definition	long definition	citation examples
Method		
Camera‐trap/Video camera	Use of camera traps or video cameras point on mistletoe plants to record interactions with animals	Amico *et al*. (2011); Mellado & Zamora (2014)
Fruit marking for removal estimates (indirect)	Indirect measure of seed removal/dispersal (*e.g*., number or proportion of marked fruits removed)	Bach & Kelly (2004); Magrach *et al*. (2013)
Focal‐plant	Use of binoculars or spotting scopes to record interactions between animals and selected mistletoes near fruit‐bearing plants	Guerra & Marini (2002); Fadini *et al*. (2010)
Active search for frugivores	Use of binoculars or spotting scopes to search actively for animals consuming mistletoe fruits in the field	Reid (1989); Overton (1996)
Tracking animals with radio/GPS	Use of radio tracking or GPS devices to track animals in the field	Ward & Paton (2007); Rawsthorne *et al*. (2011)
Collect seeds from the field or from trapped/caged animals	Collect seeds recently dispersed in the field or from trapped/caged animals for using in germination experiments	Murphy *et al*. (1993); Yan (1993)
Search for naturally dispersed seeds/seedlings	Search for seeds or seedlings naturally dispersed in potential deposition sites such as tree branches	Reid (1989); Medel *et al*. (2004)
Plant seeds manually	Plant sticky seeds manually on tree branches after their removal from the epicarp	Fadini *et al*. (2010); Maruyama *et al*. (2012)
GPT	*Gut processing time*: Time elapsed from when the animal ingests the seed until it is eliminated in its faeces or through regurgitation. Here, we used GPT as a method because it was used to obtain the seed shadow	Green *et al*. (2009); Lara *et al*. (2009)
Response variable		
Number of visits	Number of visits recorded for each animal species	Medel *et al*. (2004); Montaño‐Centellas (2013)
Number of fruits removed	Number of fruits removed by each animal species	Bach & Kelly (2004); Fontúrbel *et al*. (2017)
Movement distances	Visual estimates of the distances travelled by animals (usually in flight) from a mistletoe with fruits to potential seed deposition sites	Overton (1996); Green *et al*. (2009)
Feeding behaviour	Feeding behaviour and/or deposition behaviour (detailed handling techniques, mode of seed deposition, bird perch preferences, *etc*.)	Guerra & Marini (2002); Mellado & Zamora (2014)
Seed rain	Some kind of seed deposition pattern (*i.e*., seed rain, a modification of Wang & Smith 2002 definition)	Aukema (2004); Luo *et al*. (2016)
% Seed germination/seedling establishment of seeds collected from the field	Percentage of seed germination or seedling establishment from seeds obtained in the field or that have passed through the digestive tract of trapped or caged animals	Okubamichael *et al*. (2011); Ramírez & Ornelas (2012)
% Germination/establishment of seeds on tree branches	Percent of seed germination/survivorship/seedling establishment of seeds naturally deposited by animals or manually planted on tree branches	Davidar (1983); Roxburgh (2007)
Seed shadow	Projected seed shadow, generally using information obtained from movement distances and GPT (Wang & Smith 2002, with modifications)	Ward & Paton (2007); Rawsthorne *et al*. (2011)

## DISCUSSION

In this study, we reviewed research on mistletoe frugivory and seed dispersal from a plant–centred perspective. We highlighted the main taxonomic, geographic, and methodological gaps, and to conclude we present a brief overview of the main challenges and opportunities to guide future studies.

### The number of studies was proportional to the number of species per genus

*Psittacanthus* and *Phoradendron*, the most species diverse mistletoe genera, occur in the Neotropical region, and develop contrasting interactions with their seed dispersers. *Psittacanthus* produce large and lipid rich fruits (López de Buen & Ornelas 2001), while *Phoradendron* produces small, watery fruits that are rich in sugar (Restrepo 1987). *Psittacanthus* fruits are ingested whole and are defecated or regurgitated by birds of the families Tyrannidae, Ptiliogonatidae, Mimidae, or Bombycilidae (Overton 1994; López‐de Buen & Ornelas 1999; Fadini *et al*. 2010), while fruits of *Phoradendron* are peeled‐off before ingestion and are defecated generally by Euphonias or Chlorophonias (Davidar 1983; Cazetta & Galetti 2007; but see Restrepo *et al*. 2002). Although *P. schiedeanus* (Cham. & Schltdl.) G. Don was one of the most extensively studied species, the theoretical framework developed through the study of *P. sonorae* (S. Watson) Kuijt in the Sonoran Desert – using a metapopulation approach to explain mistletoe distribution and spread (*i.e*., Overton 1994) – was of great significance. It inspired new research on other mistletoe species (Fadini & Cintra 2015), as well as on other phorophyte‐dependent plants (*e.g*., Snäll *et al*. 2003; Löbel *et al*. 2006).

Although the most numerous mistletoe genera mentioned above have been studied, many others, such as *Helixanthera* Lour. (44 spp., occurring in Africa, Asia, and the Pacific), *Macrosolen* (Blume) Rchb. (39 spp., in Asia), and *Aetanthus* (Eichler) Engl. (18 spp., in South America), have never been studied from the perspective of frugivory and seed dispersal. Several monotypic genera have not been studied either and include the following but are not restricted to *Benthamina* Tiegh. (in Australasia); *Berhautia* Balle and *Emelianthe* Danser (in Africa); *Lampas* Danser and *Loxanthera* (Blume) Blume (in Asia); and *Notanthera* G. Don and *Desmaria* Tiegh. (in South America).

### The ‘bizarre’ adaptations were studied the most

The concentration of studies on bizarre adaptations – eloquently described by Thompson (1994) – seems to have driven the study of two congeneric species of mistletoe, *Tristerix aphyllus* Tiegh. ex Barlow & Wiens and *T. corymbosus*. The first species is a leafless, endoparasitic mistletoe, adapted to live in the Chilean semi‐deserts, nurturing from cacti and coming across to outside the host only when flowering or fruiting (Mauseth *et al*. 1984). The seeds of *T. aphyllus* are defecated by Mockingbirds (*Mimus thenca*, Mimidae) and produce the longest known mistletoe radicle, in order to overcome a protective spiny layer (Martínez del Rio *et al*. 1995). The simplicity of the system, formed basically by one mistletoe, two hosts, and one seed disperser (or seed vector), and the ease of sampling, make this species ideal for studying the transmission of parasites more generally (Martínez del Rio *et al*. 1995, 1996; Medel *et al*. 2004). This has stimulated new studies involving other mistletoe species and refined the terminology which was appropriated from parasitological studies, such as ‘vector’ instead of ‘disperser’, ‘prevalence’ instead of ‘proportion of sites occupied’, and ‘host’ instead of ‘microsite’ (Aukema & Martínez del Rio 2002a, 2002b; Roxburgh & Nicolson 2008). The system is so curious that it deserved a full episode with almost 1.2 million views to date in The Green Planet BBC Series (BBC Studios Natural History Unit 2022).

**Fig. 4:**
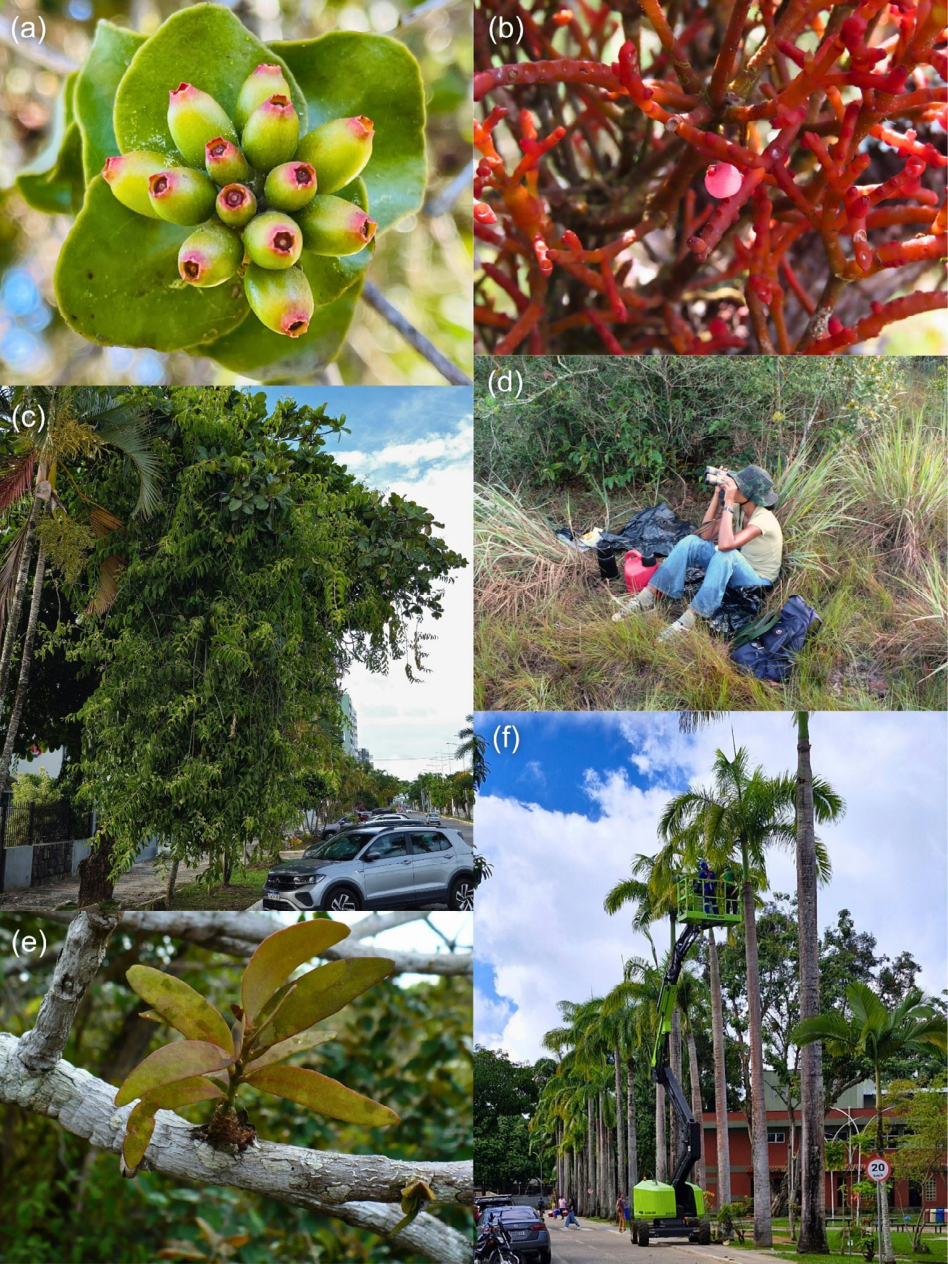
Patterns found, challenges and opportunities for mistletoe–frugivore studies: (a) The green fruits of *Tristerix corymbosus* (Loranthaceae), the most studied mistletoe species, in its southernmost distribution in Chile and Argentina. (b) *Dendrophthora fastigiata* Kuijt (Santalaceae), a red‐listed species confined to the mountain forests of South America, Colombia. (c) A mistletoe of the genus *Struthanthus* (Loranthaceae), common in urban areas of South America, forming a dense green carpet in the crown of the exotic tree *Terminalia catappa* L., Brazil. (d) The plant‐focal method is the most used to record mistletoe–animal interactions. Here, a student observing the mistletoe *Psittacanthus plagiophyllus* Eich. (Loranthaceae) in an Amazonian savanna, Brazil. (e) Mistletoes infections can be easily spotted in the tree branches and could be readily marked for population studies. Here, two infections of the mistletoe *P. plagiophyllus* in distinct ontogenetical stages, a juvenile (left) and a seedling (right). (f) Reaching the canopy of trees is a big challenge in tropical rainforests to conduct mistletoe–frugivore studies. The use of mobile aerial work platforms (MAWP) could help solve this challenge. Here, a MAWP is used for pruning a palm tree. Photo credits: Juan Camilo Muñoz (b); the authors (all others).

The second species, *Tristerix corymbosus*, is the most studied one, and is no less fascinating. It is the only known mistletoe species dispersed exclusively by a marsupial, the relictual genus *Dromiciops* spp., on its southernmost range in Chile and Argentina (Amico & Aizen 2000). The green mature fruits of *T. corymbosus* (Fig. 4a) are deposited by defecation on host branches in the understory of the Temperate Forest after being peeled‐off by *Dromiciops*, while the yellow fruits in the northernmost range (*i.e*., in the Chilean matorral) are ingested whole and dispersed by the Chilean Mockingbird (*Mimus thenca*, Mimidae), the White‐crested Elaenia (*Elaenia albiceps*, Tyrannidae), and the Austral Thrush (*Turdus falcklandii*, Turdidae). In the South American temperate forests, Amico & Aizen (2000) conclude that the marsupial is an efficient seed disperser, with over 90% of seeds passing intact through its gut and just 10% falling to the ground. That first study catalysed many others, now considering another interesting feature: the species presents yellow fruits and is bird dispersed in the northernmost range of its distribution, which generates comparative studies (Amico *et al*. 2011; Martin‐Albarracin & Amico 2024). Recently, *T. corymbosus* has received increased interest due to its potential role as a keystone species in its southernmost range, giving rise to a new research line that seeks to understand how climate change affects mistletoe, their mutualists, and an entire network of interactions through cascading effects (Fontúrbel *et al*. 2018, 2021).

### Tropical rainforest was understudied, but so was the northern temperate forests

Watson (2004) pointed out that most studies on mistletoe had been conducted in temperate forests, woodlands, semi‐arid scrublands, and savannas, while tropical mistletoe‐rich regions remained understudied. Twenty years later, we found that about half of the mistletoe species studied for frugivory and seed dispersal, and of the studies themselves, were conducted in temperate forests. However, studies in tropical rainforests are still scarce. We found only one carried out in a semi‐natural tropical rainforest in Xishuangbanna, China (Luo *et al*. 2016; see also Rist *et al*. 2011). This scarcity of studies is probably related to the difficulty of reaching the canopy of hyperdiverse tropical rainforests, which average between 35 and 40 m in height. Unfortunately, this is not restricted to frugivory or seed dispersal of mistletoes, but to mistletoe ecological studies (see Lira *et al*. 2017) and for multiple ecological processes occurring in the canopy in general.

Although the scarcity of studies in tropical forests may be related to tree accessibility, this does not explain the rarity of studies in the temperate north. We did not find a single study in the United Kingdom, where some of the classic studies on mistletoe from an animal perspective began (Snow & Snow 1984). Additionally, the only study conducted with *Viscum album* (a very abundant and widespread mistletoe across Europe) in a Mediterranean pine forest indicates that the most effective seed dispersers are generalists with a broad diet, such as *Turdus viscivorus*, although they waste many seeds by perching on thicker branches where the mistletoe cannot penetrate and therefore dries out during dry summers (Mellado & Zamora 2014). Detailed studies of frugivory and seed dispersal from the plant perspective may offer a mechanistic explanation for the recent expansion and intensification of true mistletoe (*Viscum album*) in Europe (Varga *et al*. 2014; Krasylenko *et al*. 2020).

### Studies conducted in multiple sites or with multiple species were particularly scarce

There were few studies conducted in two or more study sites or that compared mistletoes of different species. On one hand, comparative studies of the same mistletoe species in different study sites can shed light on several interesting aspects such as host‐switching mediated by seed dispersers or variation to fruit colour adaptation mediated by seed dispersers. For instance, Amico *et al*. (2011) and Martin‐Albarracin & Amico (2024) presented results of geographical variation and fruit consumption of *T. corymbosus* at 22 study sites, in each of two different biomes with contrasting seed dispersers, the Chilean Matorral (n = 8) and the South American Temperate Forests (n = 14). They concluded that the matching between fruit colour and disperser type is a consequence of ecological fitting rather than on coevolutionary processes, and that the two groups of seed dispersers (birds and *Dromiciops* spp., respectively) were indeed very effective. On the other hand, comparative studies of seed dispersal of different mistletoe species in the same study site could help to understand the role of specialists and generalists in contrasting seed dispersal systems (*e.g*., different study species or families), or how seed dispersers systems mediate mistletoe‐host specificity. For instance, Montaño‐Centellas (2013) compared the seed dispersal effectiveness of two distinct sympatric mistletoe species (*Phthirusa retroflexa* (=*Passovia* sp.) and *Struthanthus acuminatus*) dispersed basically by the same bird species. She concluded that seed dispersers performed different roles for the distinct mistletoe species, demonstrating the complementarity of seed dispersal effectiveness landscapes (SDE) formed by seed dispersers that can either deposit seeds very close to infected hosts with higher chances of seed germination, or far, with low chances of germination but able to colonize new habitats. In another interesting study, Díaz Infante *et al*. (2016) investigated reproductive isolation in two *Psittacanthus* species (*P. auriculatus* and *P. calyculatus*) growing in sympatry and allopatry. They showed that the importance of some key seed dispersers changed in response to the presence of the other mistletoe species, and suggested that reproductive isolation of the two species when in sympatry may be mediated by host and seed disperser separation rather than pollinator separation.

### There were no studies of threatened species

Fifty‐eight mistletoe species are listed as threatened worldwide (Fadini *et al*. 2024), but none of them have been studied from the frugivory perspective. Many others may also be endangered due to habitat destruction, but are not officially listed (D. Nickrent, personal communication). Most of these species have a narrow distribution, restricted to mountainous forested regions in the tropics (Fig. 4b) that are the most threatened due to climate change (Vásquez‐Aguilar *et al*. 2025). It is unclear whether these threatened mistletoes are dispersed by congener species of common frugivores present in the lowlands, but *Tristerix* spp., which are dispersed by *Mimus*, *Elaenia*, and *Turdus* (Amico *et al*. 2011) in the lowlands, are dispersed by the White‐cheeked Cotinga (*Zaratornis stresemani*, Cotingidae) and the Red‐crested Cotinga (*Ampelion rubrocristatus*, Cotingidae) in the Peruvian Andes (Parker 1981; Gil 2010). Despite these observations, very few quantitative studies have been made on seed dispersal of *Tristerix* or of any other mistletoe species in the tropical mountain forests above 2.000 m (but see Díaz Infante *et al*. 2016), especially in Africa and Asia.

### Studies in urban areas were scarce

Mistletoes are omnipresent in world urban areas (Krasylenko *et al*. 2020; Menezes *et al*. 2023). However, just one study occurring in urban areas was found in our review (Maruyama *et al*. 2012). Urban areas represent new niches for mistletoes (Fajardo *et al*. 2025), generally presenting impoverished seed disperser faunas and monodominant stands of hosts. In South America, several tree species have been introduced from Europe, Australia, or Asia, providing a banquet for native mistletoe, which spreads through their canopies, sometimes forming light green carpets that impede photosynthesis and weaken the host plants (Fig. 4c). Simultaneously, mistletoe species that were absent from some urban areas have been reintroduced (Watson *et al*. 2023). Despite this, the role of seed dispersers in the intensification or production of new infections in urban areas is still poorly understood in both cases.

### Methods and response variables, the least and the most used

Almost half of the studies have measured at least one variable related to both the quality and quantity component of dispersal of the Seed Dispersal Effectiveness framework simultaneously (SDE *sensu* Schupp 1993; Schupp *et al*. 2010), even if SDE was explicitly framed as such in only a few cases (*e.g*., Montaño‐Centellas 2013; Mellado & Zamora 2014; Fontúrbel *et al*. 2017). The quantity component has frequently been assessed using classical methods such as the ‘plant‐focal’, which consists of standing near a fruiting plant and quantifying the number of frugivore visits and the number of fruits removed per visit (Fig. 4d). The number of studies using this method is proportionally much higher than the number of studies that have used camera traps or video cameras, which have been employed over the last 10–15 years, mainly to quantify visits of *Dromiciops* to *T. corymbosus* (*e.g*., Amico *et al*. 2011; Fontúrbel *et al*. 2017).

Searching for seeds or seedlings at deposition sites has been the most widely used method to assess the quality component (Fig. 4e), as it allows measurement of both seed rain and the monitoring of germination or establishment of newly dispersed seeds (*e.g*., Reid 1989; Medel *et al*. 2004). However, if the mistletoe species studied has many dispersers, it is difficult to identify the disperser species from the seed found on the tree branch, preventing the calculation of dispersal effectiveness. The second most common way to assess the quality component has been through germination experiments using seeds collected in the field or directly from captured animals (*e.g*., Murphy *et al*. 1993; Yan 1993). Nevertheless, offering seeds to birds in captivity to quantify not only germinability but also GPT has been rarely used, especially in tropical regions (but see Okubamichael *et al*. 2011). This lack of detailed studies on fruit processing, combined with the limited use of methods to measure disperser movement, has resulted in few studies estimating seed shadow (*e.g*., Ward & Paton 2007; Rawsthorne *et al*. 2011), a key variable for evaluating the role of dispersers in long‐distance seed dispersal (Wang & Smith 2002).

### Challenges and opportunities

First, *taxonomic*: Research effort has concentrated on the most diverse genera, frequently on common species of wide distribution such as *Tristerix corymbosus*, *Phoradendron californicum*, and *Psittacanthus schiedeanus*. Therefore, new studies should prioritize the rarer species, especially those that are threatened and endemic. Rarer species may be limited by dispersal, recruitment, or both (Wang & Smith 2002). The use of seed addition experiments (Turnbull *et al*. 2000) in different hosts and under distinct environmental conditions may uncover the mechanisms behind rarity, aiding in their conservation. Candidate mistletoe species with restricted distribution and high conservation concern have already been identified elsewhere (Fadini *et al*. 2024), but many nationally or regionally threatened species should be studied as well. Another taxonomic limitation and an opportunity for new studies regards monotypic genera; 10 out of 13 genera have never been studied. These species are phylogenetically singular, and in some cases have been differentiated much earlier than their counterparts (see Liu *et al*. 2018). For instance, it is unclear whether these mistletoes are dispersed by the same animals compared with non‐monotypic sympatric species of more recent radiation (but see Ladley & Kelly 1996). *Berhautia* (*i.e., B. senegalenses*), that occurs in Senegal and Gambia (Africa) is certainly the monotypic genus with the most restricted distribution (Polhill & Wiens 1998) of all and should be studied as a key priority.

Second, *geographic*: The study of mistletoe frugivory and seed dispersal in tropical rainforests remains a major gap that extends to a methodological issue (see below). Simply, interactions are difficult to record and hosts are difficult to assess in closed‐canopy, tall tropical rainforests. Recently, Zhu *et al*. (2021) and Rossi *et al*. (2025) closed this gap, recording interactions between arboreal animals and trees in the canopy using camera traps. However, cameras were deployed at heights of 8–15 m, while mistletoe infections in mature tropical rainforests frequently occur on the slender branches of trees with >30 m or taller (Lira *et al*. 2017). Therefore, access to tree canopies is crucial and will be discussed further in the next topic.

Meanwhile, recording mistletoe–animal interactions in tropical forests is fundamental for answering a long‐lasting hypothesis, that mistletoes are ‘keystone resources in forests …providing important resources for a broad range of taxa and determining local diversities in these habitats’ (Watson 2001). For example, during the screening process, we found articles documenting mistletoe fruits in the diet of tropical monkeys (*e.g*., Norconk & Conklin‐Brittain 2016), lemurs (ZSE *et al*. 2025), and marsupials (Camargo *et al*. 2011). However, it is not clear what are their roles in seed dispersal of mistletoes in comparison to birds. Are they frequent visitors to mistletoe fruiting plants? Do they deposit seeds intact? Do they deposit seeds in the appropriate hosts and branches? How far do they move the seeds after ingesting the mistletoe fruits? These and other questions can be answered with dedicated camera‐trap studies. Another geographical gap concerns north temperate forests and woodlands, especially *Viscum album* and *Loranthus europaeus*, the two first mistletoe species formally described. Recent projections of their potential spread using MAXENT tools (one of the most popular tools for species distribution and environmental niche modelling) in pine (Walas *et al*. 2022) and oak forests (Baranowska *et al*. 2025), may renew interest in studies of seed dispersal from the plant perspective. While bioclimatic niche models (in MAXENT) provide phenomenological explanations for the spread, the obligate interaction between mistletoes and birds in Europe (Mellado & Zamora 2014; Krasylenko *et al*. 2020) may provide a process‐based, mechanistic models of spread grounded in disperser behaviour and plant traits (Nathan & Muller‐Landau 2000). Finally, the last geographical gap relates to studies of mistletoe–frugivore interactions in urban areas. Urban areas are floristically simpler than forests or woodlands and provide ideal systems for studying frugivory and seed dispersal. Combining plant‐focal observations to identify key seed dispersers with the use of animal tracking tools, could help predictive models to understand bird movement and seed deposition in urban trees (Morales & Morán López 2022), helping anticipate excessive infections or, conversely, promote mistletoe establishment where it contributes positively to urban biodiversity. Moreover, conducting research in urban areas is a great opportunity to bring birds and mistletoe closer to people's daily lives, demystifying their role in cities (Watson *et al*. 2023).

Third, *methodological*: many studies have used methods that link seed disperser activities to early stages of mistletoe recruitment. However, a clear link between seed dispersers and mistletoe population dynamics is still missing. For example, no study has evaluated the contribution of seed dispersers to the finite rate of population increase (using lambda – λ as a proxy) of mistletoes (see Godínez‐Alvarez *et al*. 2002 for an example with cactus). Mistletoes offer an easy way to test microsite suitability, and many studies have experimentally planted seeds on tree branches (Fadini *et al*. 2010; Maruyama *et al*. 2012). Yet, the studies ended once seedlings became established (likely coinciding with the duration of research grants). Long‐term demographic monitoring is therefore urgently needed, and we acknowledge that established researchers (ourselves included) bear responsibility for sustaining such efforts.

Another important gap, and an opportunity for future studies, relates animal movements to the spatial distribution of mistletoe seeds (and infections). There is an open debate about the role of dietary specialist *versus* generalist birds on mistletoe seed dispersal (Carlo & Aukema 2005; Watson 2013). Currently, specialists are viewed as self‐gardeners (Watson & Rawsthorne 2013), limiting seed dispersal to adult neighbourhoods, while generalists are thought to move seeds farther. However, this hypothesis was corroborated in only two studies conducted in simplified systems of Australian savannas, one with a dietary specialist bird (Ward & Paton 2007) and one with a dietary generalist bird (Rawsthorne *et al*. 2011), combining gut processing times (GPT) and movement rates to estimate seed shadow of a mistletoe species (*Amyema quandang*). How this translates to hyperdiverse tropical regions is not yet clear (but see Watson 2013). Given the recent advances in animal tracking using low‐cost and low‐energy demanding tools tested in urban birds (*e.g*., Farine *et al*. 2024), we propose that urban areas should be excellent arenas for testing the specialist–generalist hypothesis, especially in cities with at least one mistletoe generalist and one specialist bird (Tip: The South American genus *Struthanthus* offer an ideal study model, *e.g*., Guerra & Marini 2002). In parallel, GPT data should be collected for more species, especially for small mistletoe specialists such as *Euphonia*, *Nesotriccus*, and *Phyllomyias*, and generalists such as *Elaenia*, *Myiozetetes*, and *Pitangus* (Watson 2025), that abound in South American tree‐lined cities.

Finally, we believe that securing safe access to the canopy of the tallest trees to equip the fruiting infections with camera traps, conduct seed inoculation experiments, search for newly dispersed seeds, among others, will be the greatest advance in the study of mistletoe–frugivore interactions ever seen. We further predict that the use of mobile aerial work platforms (MAWP) (Fig. 4f) will revolutionize the study of mistletoe frugivory and seed dispersal, as well as unmanned aerial vehicles did for spatial ecology (Anderson & Gaston 2013). Methods that permit the access ‘in person’ to tree canopies can be very expensive (*e.g*., tree cranes), labor‐intensive (*e.g*., single rope techniques), or spatially limited (*e.g*., walkways platforms) (Lowman 2021). The use of MAWP, on the other hand, can be cost‐effective in the medium term and would permit access to multiple trees in a single day. However, logistically the studies would be limited to trees located close to forest borders, which is not a problem in some forest conditions. For example, frugivore community and frugivore–plant interactions are similar between undisturbed and logged forests that used low‐impact practices in the Amazon (Rossi *et al*. 2025). Therefore, the frugivore communities may be close to the original set of potential frugivores that would interact with mistletoe eventually sampled with MAWP.

## CONCLUDING REMARKS

Mistletoe–frugivore interactions offer excellent study models for exploring a different suite of ecological theories. They are easy to spot, easy to measure, and provide reliable material for dedicated studies. Despite this, the study of interactions between mistletoes and frugivores should not be taken for granted, as many groups of species, regions, and biomes have been poorly studied so far. Additionally, we believe that a big picture is still missing. Ultimately, what role do animals (and birds in particular) play in the population dynamics of mistletoe? We also believe that many old and new unanswered questions could be clarified using modern and appropriate methods to assess the interactions between mistletoe and frugivores, especially in tropical forests. For instance: Are mistletoes actually key food resources for animals (not just birds) in tropical rainforests? What is the contribution of seed dispersers to the finite population growth rate of mistletoes? What is the relative contribution of generalist and specialist frugivores on seed dispersal effectiveness of mistletoes? Among others.

## AUTHOR CONTRIBUTIONS

RFF conceived the ideas, designed the study, and conducted the review; MAP and EC assisted in quality control of the review; RFF led the writing of the manuscript. MAP, EC, and FEF critically reviewed the drafts of the manuscript. All authors gave final approval for publication.

## CONFLICT OF INTEREST

The authors have no competing interests to declare that are relevant to the content of this article.

## Supporting information


**Fig. S1.** Flowchart with article selection processes and number of studies for.
**Fig. S2.** Number of published papers per year in the Web of Knowledge and Scopus databases (searched on 24 June 2025), using a combination of search terms regarding to mistletoe frugivory and seed dispersal. See text for details.
**Table S1.** Inclusion and exclusion criteria for articles selection.

## Data Availability

The review process and data were deposited in the open access FigShare Repository at https://doi.org/10.6084/m9.figshare.31255543 (Fadini et al., 2026).
