# Supplementary material for: A global review of mistletoe frugivory and seed dispersal: The plant perspective
Source: Plant Biol (Stuttg). 2026 Jun 29;28(5):1303–14. doi: 10.1111/plb.70252 (PMC13358654; doi:10.1111/plb.70252)
Supplement: Supplementary file 1 — Fig. S1. Flowchart with article selection processes and number of studies for. Fig. S2. Number of published papers per year in the Web of Knowledge and Scopus databases (searched on 24 June 2025), using a combination of search terms regarding to mistletoe frugivory and seed dispersal. See text for details. Table S1. Inclusion and exclusion criteria for articles selection. [file PLB-28-1303-s001.docx]

**Supplementary material from “A global review of mistletoe frugivory and seed dispersal: the plant perspective”**

Rodrigo F. Fadini^1,2,3,4^, Marco A. Pizo^5^, Francisco E. Fontúrbel^3,6,7^, Eliana Cazetta^2,4,8^

^1^ Laboratório de Ecologia e Conservação (LabECon), Instituto de Biodiversidade e Florestas, Universidade Federal do Oeste do Pará, Santarém, Brazil

^2^ Laboratório de Ecologia Aplicada à Conservação (LEAC), Universidade Estadual de Santa Cruz, Ilhéus, Brazil

^3^ Grupo de Estudos em Plantas Parasitas, Universidade Estadual do Sudoeste da Bahia, Vitória da Conquista, Brazil

^4^ DISPERSE: Rede de Pesquisa sobre Frugivoria e Dispersão de Sementes, Universidade Estadual de Santa Cruz, Ilhéus, Brazil

^5^ Laboratório de Ecologia de Aves, Universidade Estadual Paulista Júlio de Mesquista Filho, Rio Claro, Brazil

^6^ Instituto de Biología, Pontificia Universidad Católica de Valparaíso, Valparaíso, Chile

^7^ Millennium Nucleus of Patagonian Limit of Life (LiLi), Valdivia, Chile

^8^ Laboratório de Ecologia de Interações (LABINT), Universidade Estadual de Santa Cruz, Ilhéus, Brazil

^9^Author for correspondence: rodrigo.fadini@ufopa.edu.br

**Methods**

**Table S1.** Inclusion and exclusion criteria for articles selection.

| **Inclusion/Exclusion criteria** |
| --- |
| 1. We considered studies that published quantitative results on the frugivory or seed dispersal of at least one mistletoe species (e.g., anecdotal information obtained from field guides and popular science books were not considered). |
| 1. Studies that did not have the frugivore identified using any methodology from the article itself (e.g., focal observation or camera-trap recording) were included only if they had at least some quantitative measure of dispersal (e.g., fruits removed, seeds dispersed to branches). In these cases, we marked the disperser identification as "not available" (NA). |
| 1. We considered only studies published in English. |
| 1. 5. We only considered mistletoe species dispersed by endozoochory. *Arceuthobium*, *Misodendrum*, and *Korthalsella*, which are species dispersed by abiotic vectors, were excluded. |
| 1. We included only studies indexed in the Scopus and Web of Science databases. Unpublished studies and studies with secondary data (e.g., reviews) were not considered. |
| 1. Studies of root-parasitic Loranthaceae (e.g., *Atkinsonia*, *Gaiadendron*, or *Nuytsia*) or Santalaceae (e.g., *Acanthosyris*) were excluded. |
| 1. Studies that exclusively document another type of mutualistic interaction (e.g., pollination) were excluded. |

**Results**

Records identified:

Databases (n = 2)

Records: 2934

Scopus: 839

Web of Science (WoS): 2095

Records removed *before screening*:

Duplicate records removed (n = 2291)

Records removed for other reasons (n = 65)

**Identification**

Records screened

(n = 515)

Records excluded

(n = 387)

**Screening**

Reports assessed for eligibility

(n = 128)

Reports excluded: 72

Number of studies (WoS and Scopus)

(n = 56)

Number of studies from the authors (n=2)

Number of studies included in review (n = 58)

**Included**

**Figure S1.** Flowchart with article selection processes and number of studies for

each step of the review based on PRISMA-EcoEvo 1.0 protocol (O’ Dea et al., 2021).

**Figure S2.** Number of published papers per year in the Web of Knowledge and Scopus databases (searched on 24 June 2025), using a combination of search terms regarding to mistletoe frugivory and seed dispersal. See text for details.

**List of references used in this review (n = 58)**

Aizen MA (2003) Influences of animal pollination and seed dispersal on winter flowering in a temperate mistletoe. Ecology **84**: 2613–2627. <https://doi.org/10.1890/02-0521>

Amico G & Aizen MA (2000) Mistletoe seed dispersal by a marsupial. Nature **408**: 929-930. <https://doi.org/10.1038/35050170>

Amico GC, Rodriguez‐Cabal MA & Aizen MA (2011) Geographic variation in fruit colour is associated with contrasting seed disperser assemblages in a south‐Andean mistletoe. Ecography **34**: 318–326. <https://doi.org/10.1111/j.1600-0587.2010.06459.x>

Amico GC, Sasal Y, Vidal‐Russell R, Aizen MA & Morales JM (2017) Consequences of disperser behaviour for seedling establishment of a mistletoe species. *Austral Ecology* 42: 900–907. <https://doi.org/10.1111/aec.12517>

Aukema JE & Martínez del Rio C (2002a) Variation in mistletoe seed deposition: effects of intra- and interspecific host characteristics. Ecography **25**: 139–144. <https://doi.org/10.1034/j.1600-0587.2002.250202.x>

Aukema JE & Martínez del Rio C (2002b) Where does a fruit-eating bird deposit mistletoe seeds? Seed deposition patterns and an experiment. Ecology **83**: 3489–3496. [https://doi.org/10.1890/0012-9658(2002)083[3489:WDAFEB]2.0.CO;2](https://doi.org/10.1890/0012-9658(2002)083%5b3489:WDAFEB%5d2.0.CO;2)

Aukema JE (2004) Distribution and dispersal of desert mistletoe is scale-dependent, hierarchically nested. Ecography **27**: 137–144. <https://doi.org/10.1111/j.0906-7590.2004.03640.x>

Bach CE & Kelly D (2004) Effects of forest edges, fruit display size, and fruit colour on bird seed dispersal in a New Zealand mistletoe, *Alepis flavida*. New Zealand Journal of Ecology **28**: 93–103.

Botto-Mahan C, Medel R, Ginocchio R & Montenegro G (2000) Factors affecting the circular distribution of the leafless mistletoe *Tristerix aphyllus* (Loranthaceae) on the cactus *Echinopsis chilensis*. Revista Chilena de Historia Natural **73**: 525–531.

Caraballo‐Ortiz MA, González‐Castro A, Yang S, dePamphilis CW & Carlo TA (2017) Dissecting the contributions of dispersal and host properties to the local abundance of a tropical mistletoe. Journal of Ecology **105**: 1657–1667. <https://doi.org/10.1111/1365-2745.12795>

Carlo TA & Aukema JE (2005) Female‐directed dispersal and facilitation between a tropical mistletoe and a dioecious host. Ecology **86**: 3245–3251. <https://doi.org/10.1890/05-0460>

Cazetta E & Galetti M (2007) Frugivory and host specificity in the mistletoe *Phoradendron rubrum* (L.) Griseb. (Viscaceae). Brazilian Journal of Botany **30**: 345–351. <https://doi.org/10.1590/S0100-84042007000200017>

Davidar P (1983) Birds and neotropical mistletoes: effects on seedling recruitment. Oecologia **60**: 271–273. <https://doi.org/10.1007/BF00379532>

Díaz Infante S, Lara L, Arizmendi MDC, Eguiarte LE & Ornelas JF (2016) Reproductive ecology and isolation of *Psittacanthus calyculatus* and *P. auriculatus* mistletoes (Loranthaceae). PeerJ **4**: e2491. <https://doi.org/10.7717/peerj.2491>

Fadini RF, Gonçalves DCM & Reis RPF (2010) Consistency in seed-deposition patterns and the distribution of mistletoes among its host trees in an Amazonian savanna. Australian Journal of Botany **57**: 640–646. <https://doi.org/10.1071/BT09013>

Fenosoa ZSE, Brook CE, Dunham AE, Rakotomanana HF, Razafindraibe H & Ring KA (2025) Gut passage in lemurs enhances the germination of mistletoe seeds of *Bakerella gonoclada*, in a Madagascar Rainforest. African Journal of Ecology **63**: e70012. <https://doi.org/10.1111/aje.70012>

Fontúrbel FE, Lara A, Lobos D & Little C (2018) The cascade impacts of climate change could threaten key ecological interactions. Ecosphere **9**: e02485. <https://doi.org/10.1002/ecs2.2485>

Fontúrbel FE, Jordano P & Medel R (2017) Plant-animal mutualism effectiveness in native and transformed habitats: Assessing the coupled outcomes of pollination and seed dispersal. Perspectives in Plant Ecology, Evolution and Systematics **28**: 87–95. <https://doi.org/10.1016/j.ppees.2017.09.003>

García D, Rodríguez‐Cabal MA & Amico GC (2009) Seed dispersal by a frugivorous marsupial shape the spatial scale of a mistletoe population. Journal of Ecology **97**: 217–229. <https://doi.org/10.1111/j.1365-2745.2008.01470.x>

Godschalk SKB (1983) Feeding behavior of avian dispersers of mistletoe fruit in the Loskop-dam-nature-reserve, South Africa. South African Journal of Zoology **20**: 136–146.

Green AK, Ward D & Griffiths ME (2009) Directed dispersal of mistletoe (*Plicosepalus acaciae*) by Yellow-vented Bulbuls (*Pycnonotus xanthopygos*). Journal of Ornithology **150**: 167–173. <https://doi.org/10.1007/s10336-008-0331-9>

Guerra TJ & Marini MÂ (2002) Bird frugivory on *Struthanthus concinnus* (Loranthaceae) in southeastern Brazil. Ararajuba **10**: 187–192.

Guerra TJ & Pizo MA (2014) Asymmetrical dependence between a Neotropical mistletoe and its avian seed disperser. Biotropica **46**: 285–293. <https://doi.org/10.1111/btp.12112>

Hoffmann AJ, Fuentes ER, Cortes I, Liberona F & Costa V (1986) *Tristerix tetrandrus* (Loranthaceae) and its host-plants in the Chilean matorral: patterns and mechanisms. Oecologia **69**: 202–206. <https://doi.org/10.1007/BF00377622>

Ladley JJ & Kelly D (1996) Dispersal, germination and survival of New Zealand mistletoes (Loranthaceae): dependence on birds. New Zealand Journal of Ecology **20**: 69–79.

Lara C, Pérez G & Ornelas JF (2009) Provenance, guts, and fate: field and experimental evidence in a host-mistletoe-bird system. Ecoscience **16**: 399–407. <https://doi.org/10.2980/16-3-3235>

Larson DL (1996) Seed dispersal by specialist versus generalist foragers: the plant's perspective. Oikos **76**: 113–120. <https://doi.org/10.2307/3545753>

López de Buen L & Ornelas JF (1999) Frugivorous birds, host selection and the mistletoe *Psittacanthus schiedeanus*, in central Veracruz, Mexico. Journal of Tropical Ecology **15**: 329–340. <https://doi.org/10.1017/S0266467499000851>

Lucero F, Botto-Mahan C & Medel R (2014) New insights on the mistletoe *Tristerix aphyllus* (Loranthaceae): interaction with diurnal and nocturnal frugivorous species. Gayana Botanica **71**: 270–272.

Luo Y, Sui Y, Gan J & Zhang L (2016) Host compatibility interacts with seed dispersal to determine small-scale distribution of a mistletoe in Xishuangbanna, Southwest China. Journal of Plant Ecology **9**: 77–86. <https://doi.org/10.1093/jpe/rtv024>

Magrach A, Santamaría L & Larrinaga AR (2013) Forest edges show contrasting effects on an austral mistletoe due to differences in pollination and seed dispersal. Journal of Ecology **101**: 713–721. <https://doi.org/10.1111/1365-2745.12083>

Martin-Albarracin VL & Amico GC (2024) Seed dispersal of the mistletoe *Tristerix corymbosus*: a comparative study in two biomes with different animal assemblages. Plant Ecology **225**: 1209–1218. <https://doi.org/10.1007/s11258-024-01466-x>

Martínez del Rio CM, Hourdequin M, Silva A & Medel R (1995) The influence of cactus size and previous infection on bird deposition of mistletoe seeds. Australian Journal of Ecology **20**: 571–576. <https://doi.org/10.1111/j.1442-9993.1995.tb00577.x>

Martínez del Rio CM, Silva A, Medel R & Hourdequin M (1996) Seed dispersers as disease vectors: bird transmission of mistletoe seeds to plant hosts. Ecology **77**: 912–921. <https://doi.org/10.2307/2265511>

Maruyama PK, Mendes-Rodrigues C, Alves-Silva E & Cunha AF (2012) Parasites in the neighbourhood: interactions of the mistletoe *Phoradendron affine* (Viscaceae) with its dispersers and hosts in urban areas of Brazil. Flora **207**: 768–773. <https://doi.org/10.1016/j.flora.2012.08.004>

Medel R, Vergara E, Silva A & Kalin-Arroyo M (2004) Effects of vector behavior and host resistance on mistletoe aggregation. Ecology **85**: 120-126. <https://doi.org/10.1890/03-0261>

Mellado A & Zamora R (2014) Generalist birds govern the seed dispersal of a parasitic plant with strong recruitment constraints. Oecologia **176**: 139–147. <https://doi.org/10.1007/s00442-014-3013-8>

Montaño‐Centellas FA (2013) Effectiveness of mistletoe seed dispersal by tyrant flycatchers in a mixed Andean landscape. Biotropica **45**: 209–216. <https://doi.org/10.1111/j.1744-7429.2012.00909.x>

Murphy SR, Reid R, Yan Z & Venables WN (1993) Differential passage time of mistletoe fruits through the gut of honeyeaters and flowerpeckers: effects on seedling establishment. Oecologia **93**: 171–176. <https://doi.org/10.1007/BF00317667>

Okubamichael DY, Rasheed MZ, Griffiths ME & Ward D (2011) Avian consumption and seed germination of the hemiparasitic mistletoe *Agelanthus natalitius* (Loranthaceae). Journal of Ornithology **152**: 643–649. <https://doi.org/10.1007/s10336-010-0624-7>

Overton JM (1994) Dispersal and infection in mistletoe metapopulations. Journal of Ecology **82**: 711–723. <https://doi.org/10.2307/2261437>

Overton JM (1996) Spatial autocorrelation and dispersal in mistletoes: field and simulation results. Vegetatio **125**: 83–98. <https://doi.org/10.1007/BF00045207>

Raji IA, Chaskda AA, Manu SA & Downs CT (2021) Bird species use of *Tapinanthus dodoneifolius* mistletoes parasitising African locust bean trees *Parkia biglobosa* in Amurum Forest Reserve, Nigeria. Journal of Ornithology **162**: 1129–1140. <https://doi.org/10.1007/s10336-021-01890-0>

Ramírez MM & Ornelas JF (2009) Germination of *Psittacanthus schiedeanus* (mistletoe) seeds after passage through the gut of Cedar Waxwings and Grey Silky-flycatchers. The Journal of the Torrey Botanical Society **136**: 322–331. <https://doi.org/10.3159/09-RA-023.1>

Ramírez MM & Ornelas JF (2012) Cross-infection experiments of *Psittacanthus schiedeanus*: effects of host provenance, gut passage, and host fate on mistletoe seedling survival. Plant Disease **96**: 780-787. <https://doi.org/10.1094/PDIS-06-11-0509>

Rawsthorne J, Watson DM & Roshier DA (2011) Implications of movement patterns of a dietary generalist for mistletoe seed dispersal. Austral Ecology **36**: 650–655. <https://doi.org/10.1111/j.1442-9993.2010.02200.x>

Rawsthorne J, Watson DM & Roshier DA (2012) The restricted seed rain of a mistletoe specialist. Journal of Avian Biology **43**: 9–14. <https://doi.org/10.1111/j.1600-048X.2011.05515.x>

Reid N (1989) Dispersal of mistletoes by honeyeaters and flowerpeckers: components of seed dispersal quality. Ecology **70**: 137–145. <https://doi.org/10.2307/1938420>

Rodríguez-Cabal MA, Aizen AM & Novaro AJ (2007) Habitat fragmentation disrupts a plant-disperser mutualism in the temperate forest of South America. Biological Conservation **139**: 195–202. <https://doi.org/10.1016/j.biocon.2007.06.014>

Roxburgh L & Nicolson SW (2005) Patterns of host use in two African mistletoes: the importance of mistletoe-host compatibility and avian disperser behaviour. Functional Ecology **19**: 865–873. <https://doi.org/10.1111/j.1365-2435.2005.01036.x>

Roxburgh L & Nicolson SW (2008) Differential dispersal and survival of an African mistletoe: does host size matter? Plant Ecology **195**: 21–31. <https://doi.org/10.1007/s11258-007-9295-8>

Roxburgh L (2007) The effect of gut processing on the quality of mistletoe seed dispersal. Journal of Tropical Ecology **23***:* 377–380. <https://doi.org/10.1017/S0266467407004014>

Soto-Gamboa M & Bozinovic F (2002) Fruit-disperser interaction in a mistletoe-bird system: a comparison of two mechanisms of fruits processing on seed germination. Plant Ecology **159**: 171–174. <https://doi.org/10.1023/A:1015514707286>

Tiribelli F, Amico GC, Sasal Y & Morales JM (2017) The effect of spatial context and plant characteristics on fruit removal. Acta Oecologica **82**: 69–74. <http://dx.doi.org/10.1016/j.actao.2017.06.002>

Ward MJ & Paton DC (2007) Predicting mistletoe seed shadow and patterns of seed rain from movements of the mistletoebird, *Dicaeum hirundinaceum*. Austral Ecology **32**: 113–121. <https://doi.org/10.1111/j.1442-9993.2006.01668.x>

Watson DM (2013) The relative contribution of specialists and generalists to mistletoe dispersal: insights from a Neotropical rain forest. Biotropica **45**: 195–202. <https://doi.org/10.1111/j.1744-7429.2012.00905.x>

Yan Z (1993) Seed dispersal of *Amyema preissii* and *Lysiana exocarpi* by mistletoebirds and spiny-cheeked honeyeaters. Emu-Austral Ornithology **93**: 214–219. <https://doi.org/10.1071/MU9930214>

Zhao Y, Xu W, Sun Q, Cheng K & Zong C (2025) Mistletoe *Viscum coloratum* maintains a stable mutualistic network in temperate forest by strategic seed provisioning to avian dispersers. Global Ecology and Conservation **64**: e03944. <https://doi.org/10.1016/j.gecco.2025.e03944>
